# Genetic characteristics influence the phenotype of marine macroalga *Fucus vesiculosus* (Phaeophyceae)

**DOI:** 10.1002/ece3.9788

**Published:** 2023-01-31

**Authors:** Roxana Preston, Iván F. Rodil

**Affiliations:** ^1^ Ecosystems and Environment Research Programme, Faculty of Biological and Environmental Sciences University of Helsinki Helsinki Finland; ^2^ Tvärminne Zoological Station University of Helsinki Hanko Finland; ^3^ Department of Biology, INMAR University of Cadiz, International Campus of Excellence of the Sea (CEIMAR) Cádiz Spain

**Keywords:** clonality, Gigas effect, intraclonal variation, phenotypic plasticity, polyploidy, somatic mutations

## Abstract

Intraspecific variation is an important component of heterogeneity in biological systems that can manifest at the genotypic and phenotypic level. This study investigates the influence of genetic characteristics on the phenotype of free‐living *Fucus vesiculosus* using traditional morphological measures and microsatellite genotyping. Two sympatric morphotypes were observed to be significantly genetically and morphologically differentiated despite experiencing analogous local environmental conditions; indicating a genetic element to *F. vesiculosus* morphology. Additionally, the observed intraclonal variation established divergent morphology within some genets. This demonstrated that clonal lineages have the ability to alter morphological traits by either a plastic response or somatic mutations. We provide support for the potential occurrence of the Gigas effect (cellular/organ enlargement through genome duplication) in the *Fucus* genus, with polyploidization appearing to correlate with a general increase in the size of morphological features. Phenotypic traits, as designated by morphology within the study, of *F. vesiculosus* are partially controlled by the genetic characteristics of the thalli. This study suggests that largely asexually reproducing algal populations may have the potential to adapt to changing environmental conditions through genome changes or phenotypic plasticity.

## Graphical abstract

Using traditional morphological measures and microsatellite genotyping, the relationships between genotype and phenotype of free‐living *Fucus vesiculosus* are investigated. We determine intraspecific morphological variation within clonal lineages as well as within genetically differentiated, sympatric morphotypes. Polyploidization corresponds to an increased thallus size potentially indicating the Gigas effect.

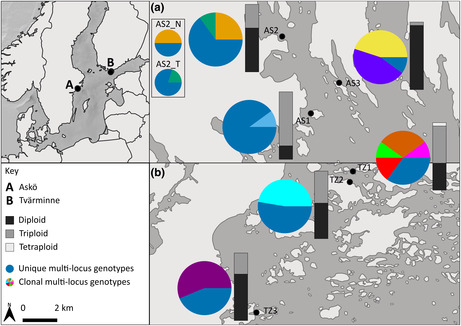

## 1 INTRODUCTION

Complexity is an important component of biological systems (Cadenasso et al., 2006). This complexity manifests as heterogeneity in the biological environment, with the scale of heterogeneity ranging through genes, individuals, and populations up to communities and ecosystems. Heterogeneity of individuals within a population can be displayed through the genotype and phenotype. Genotype represents the genetic characteristics of an individual, and is often subject to evolutionary processes (Andrews, 2010). Phenotype, the observable attributes of an individual, can represent aspects of an individual's morphology, physiology, and behavior (Sommer, 2020). Differences in genotype and phenotype characterize the intraspecific variation of a species. There is growing recognition that intraspecific variation plays an important role in biological systems (Bolnick et al., 2011; Violle et al., 2012). For example, intraspecific phenotypic variation can influence plant–herbivore interactions (Cruz‐Rivera & Friedlander, 2013), while greater intraspecific genetic variation in key species has been shown to increase the complexity of the associated food web (Barbour et al., 2016). Consequently, the genotypic and phenotypic complexity of a species can have ecosystem‐level effects. Within a species, genotypes and phenotypes are disparate. Many genotypes may encode the same phenotype, while the number of genotypes encoding each phenotype is also unevenly distributed (Catalán et al., 2018). Consequently, potential complexity is controlled by the specific biological properties of a species.

The relationship between genetic factors and phenotype is better understood in animals, plants, and yeasts (Čertner et al., 2019; Martin & Orgogozo, 2013), yet in macroalgae this relationship is less known. *Fucus* is a genus of brown algae known for high phenotypic plasticity and morphological variation (Kucera & Saunders, 2008; Powell, 1963; Wallace et al., 2004). Several form series have been described (e.g., Baker & Bohling, 1916; Kjellman, 1890; Svedelius, 1901), and taxonomic classification by traditional morphological techniques is often confounded by the complex morphological variation (Kucera & Saunders, 2008). For *Fucus vesiculosus* L., trends between morphology and environmental factors are well described (Bäck, 1993; Barboza et al., 2019; Kalvas & Kautsky, 1998; Ruuskanen & Bäck, 2002, Ruuskanen & Bäck, 1999). The species has also been noted to display phenotypic plasticity in response to herbivory (Haavisto et al., 2010; Peckol et al., 1996; Rohde et al., 2004; Rohde & Wahl, 2008) and environmental stimuli (Peckol et al., 1996). Although typically associated with hard substratum as an attached form, *F. vesiculosus* can also be frequently found in a free‐living form lying unattached on any substratum. This form is particularly common throughout the Baltic Sea (Bauch, 1954; Luther, 1981; Luther et al., 1975; Meyer et al., 2019; Preston, Seppä, et al., 2022). Free‐living populations are known to display vast morphological variation, as determined by several form series (Kjellman, 1890; Svedelius, 1901). Here, we investigate the role of genetic factors on phenotypic variation in several free‐living *F. vesiculosus* populations within the Baltic Sea. We focus on morphological divergence and how genotypic factors influence this. Atypically for *F. vesiculosus*, the Baltic Sea distribution demonstrates facultative asexuality (Ardehed et al., 2016; Johannesson et al., 2011; Pereyra et al., 2013; Tatarenkov et al., 2005). Asexual reproduction, presumably by means of fragmentation and/or adventitious branches, is particularly pervasive within the free‐living form, although the prevalence of clonality is highly variable among populations (Preston, Blomster, et al., 2022). Accordingly, free‐living populations provide an ideal study system as they can consist of varying proportions of clones, either from single or multiple lineages, and unique multilocus genotypes (MLGs) (Preston, Blomster, et al., 2022). Each clone represents a physiological individual (ramet), collectively all ramets originate from a single zygote (genet) and are genetically identical barring somatic mutations (Harper, 1977; Jackson & Coates, 1986; Pan & Price, 2002). Consequently, the occurrence of multiple ramets can provide a novel opportunity to explore aspects of morphological traits.

Polyploidy (whole‐genome replication) can affect phenotype, as has been frequently observed in plants (Levin, 1983; Soltis et al., 2014). In plants, polyploidy is typically associated with larger cell sizes compared to diploidy (Müntzing, 1936; Ramsey & Ramsey, 2014; Stebbins, 1971), which in turn correlates with larger adult sizes (Čertner et al., 2019; Te Beest et al., 2012). Unattached *Fucus* spp. are known to represent varying ploidy levels, with both allopolyploidy (hybridization between two or more related species) and autopolyploidy (multiplication of the whole genome of a single parent species) having been observed in natural populations (Coyer et al., 2006; Sjøtun et al., 2017). Recent investigations indicate that polyploidization may also occur within free‐living *F. vesiculosus* within the Baltic Sea (Preston, Blomster, et al., 2022). However, polyploidy in *Fucus* spp. has typically been associated with miniaturization of the thalli (e.g., *F. cottonii* (Sjøtun et al., 2017) and “muscoides‐like” *Fucus* (Coyer et al., 2006)). Thus, the current anecdotal observations of the correlation between ploidy and morphology in *Fucus* spp. are in opposition to the more widely accepted understanding within plants.

Algae in general are also known for high phenotypic plasticity (Lürling, 2003; Ragazzola et al., 2013; Thiriet‐Rupert et al., 2021), and *Fucus* spp. are no exception (Padilla & Savedo, 2013; Rugiu et al., 2018). By definition, phenotypic plasticity construes that one genotype has the ability to express a multitude of phenotypes in response to biotic and abiotic conditions (Bradshaw, 1965). Accordingly, species characterized by a high degree of phenotypic plasticity have the ability to express a large range of phenotypes from single genotypes. The effects of phenotypic plasticity are largely underappreciated when it comes to diversification, yet they can greatly impact intraspecific variation (Pfennig et al., 2010) including morphological aspects of an organism's phenotype (Sommer, 2020). Phenotypic plasticity can also effect ecosystems at varying levels, having a variety of direct and indirect interactions at the individual, population, and community level (Miner et al., 2005). In clonal populations, phenotypic plasticity represents an ability for a genet to adapt to diverse environmental conditions (Bruno & Edmunds, 1997; Geng et al., 2016). Whether dispersed or in close proximity, ramets experience different selective pressures as a result of varying biotic and abiotic conditions. Phenotypic plasticity can allow an adaptive response to these variable conditions in the absence of recombination, allowing the persistence of the genet. The resulting plastic responses produce intraclonal variability, creating an additional level of complexity within clonal lineages. Intraclonal variation can also be the result of genome changes during somatic growth (Santelices, 2004; Santelices & Varela, 1993). Somatic mutations, genetic changes that occur during mitosis, can be a result of internal or external environmental factors such as temperature stress, cell age, and oxidative stress (Schoen & Schultz, 2019). Often neutral or beneficial, somatic mutations may result in phenotypic changes, as seen by their frequent occurrence in commercially important plant varieties (Tilney‐Bassett, 1986). These mutations can accumulate, with the potential to be inherited by progeny or propagated in ramets. The accumulation of somatic mutations can result in genetic mosaicism, which in clonal lineages can lead to the formation of genotypically diverse, independent ramets (Gill et al., 1995). As somatic mutations are potentially heritable, there are consequences for evolutionary processes and diversification in clonal lineages that otherwise have limited recombination (Gross et al., 2012; Klekowski, 1988). Clonal lineages can therefore represent a mosaic of phenotypes due to either potentially reversible plastic responses or genetically fixed genome changes. Overall intraclonal variation can promote adaptive responses and potential genet persistence in changing environments for facultative asexual species, such as *F. vesiculosus*, as well as acting as a potential source of intraspecific complexity. As a highly plastic species with persistent clonality, we expect to observe intraclonal morphological variation within free‐living *F. vesiculosus* clonal lineages.

Overall, this study aims to examine the influence of genotype and polyploidy on morphological traits of free‐living *F. vesiculosus* at the intraspecific and intraclonal level.

## 2 MATERIALS AND METHODS

### 2.1 Sampling

**FIGURE 1:**
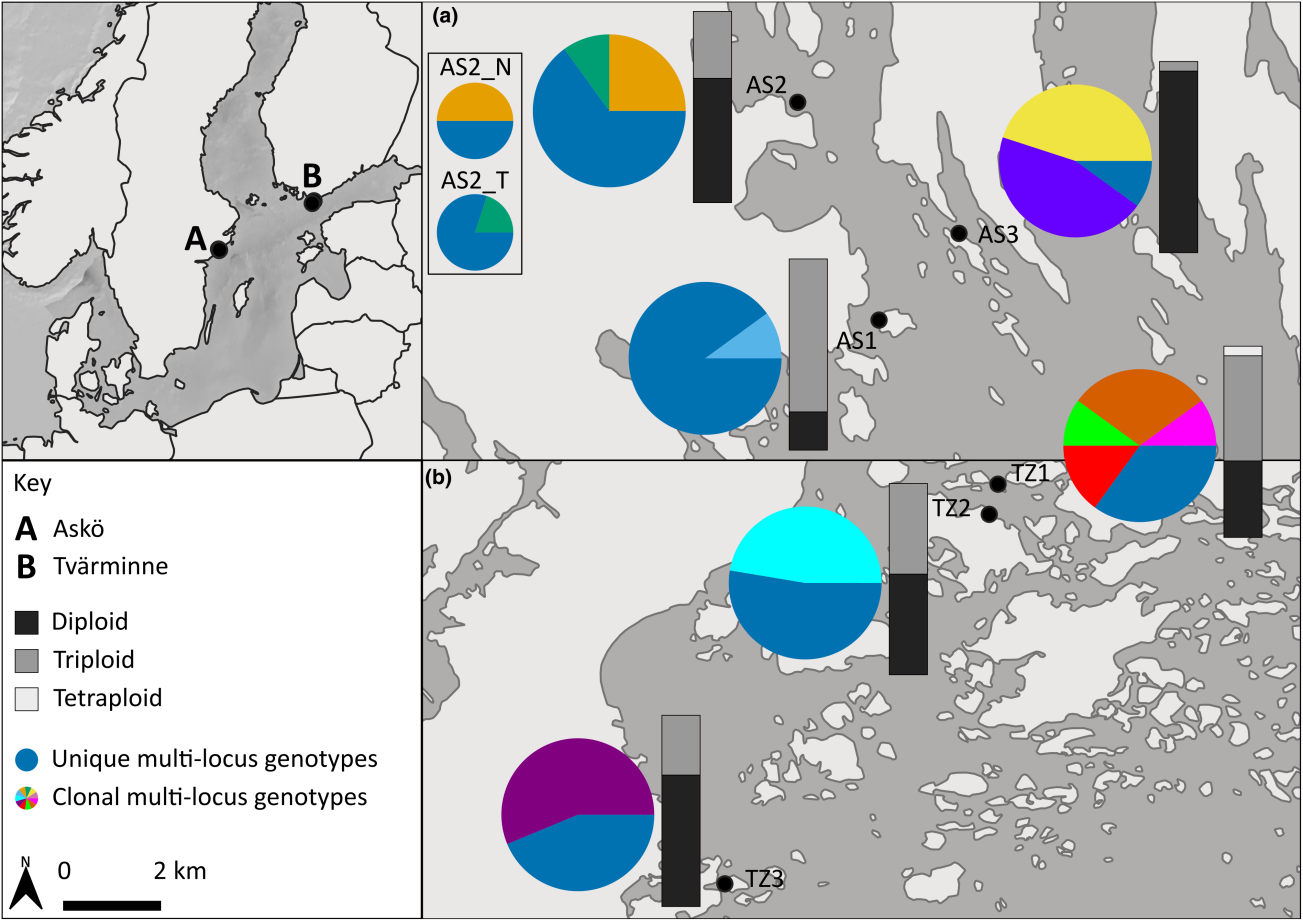
Proportions of multilocus genotypes (circles) and ploidy levels (bars) at each *Fucus vesiculosus* site. Off‐white color represents land and gray represents sea. Scale bar dictates distances in inset maps A and B. Site abbreviations: TZ, Tvärminne; AS, Askö.

Sampling was performed by SCUBA diving in June 2019 at Askö [3 sites: AS1–AS3] in the Northern Baltic Proper and Tvärminne [3 sites: TZ1–TZ3] in the Gulf of Finland (Table 1, Figure 1). All sites were within close proximity of the shore, in shallow, sheltered embayments associated with *Phragmites australis* reed beds (Data S1). The bottoms at all sites were soft, being either muddy or sandy substrata. Sampling depth ranged between 1.5 and 3.4 m, and the salinity ranged from c.5.9 to 6.1. Free‐living *F. vesiculosus* was the dominant macroalga within these locations. At sites AS1 and TZ1, the thalli were entangled within *P. australis*. At each site, four 20 × 20 cm frames were randomly placed on the seafloor with all vegetation within the frame being collected into net bags. If available, up to five *F. vesiculosus* thalli per frame (*n* = 115) were selected for further measurements. In frames with more than five thalli, all other collected thalli (excluding the selected five per frame) were discarded from further analysis. All detached thalli were treated as separate physiological individual thalli for means of morphological analysis and genotyping. If fewer than five separate thalli were found within the plot, then the maximum number of separate thalli available was chosen.

**TABLE 1:** Sample collection information.

Site	Subbasin	Region	Country	Site coordinates (Decimal degrees)	Sampling date	No. of thalli genotyped	No. of genotypes	Clonal lineages
AS1	Northern Baltic Proper	Askö	Sweden	59.910 23.381	13/06/2019	20	19	C46
AS2	Northern Baltic Proper	Askö	Sweden	59.905 23.376	13/06/2019	20	15	C50, C58
AS3	Northern Baltic Proper	Askö	Sweden	59.846 23.252	13/06/2019	20	4	C65, C66
TZ1	Gulf of Finland	Tvärminne	Finland	58.895 17.628	1/06/2019	20	11	C1, C2, C3, C4
TZ2	Gulf of Finland	Tvärminne	Finland	58.937 17.607	1/06/2019	19	11	C12
TZ3	Gulf of Finland	Tvärminne	Finland	58.909 17.660	2/06/2019	16	8	C23

### 2.2 Morphological measurements

The 115 collected thalli (max 5 per frame) underwent morphological analysis. Due to the free‐living nature of the *F. vesiculosus* thalli (i.e., lack of holdfast), many of the standard morphological measures normally used for *Fucus* spp. are inappropriate (e.g., stipe length, stipe width, and distance of dichotomies). Consequently, three standard measures not reliant on the presence of a holdfast were recorded. These measures were thallus height (cm), wet weight (g), and five repeats of thallus width (cm) per individual sample (Ruuskanen & Bäck, 1999, 2002). Thallus height was determined as the distance from oldest growth to the longest apical tip, while thallus width was the width across the thallus 5 cm from the apical tip. Site AS2 displayed two distinct morphotypes coexisting with no perceivable abiotic barriers (Data S2). Within this study, morphotype was defined as a group of thalli with similar morphology using our defined morphological measures. Samples were taken from both morphotypes and defined as AS2_N (narrow morphotype) and AS2_T (typical morphotype). The distinction between the morphotypes was evident through visual inspection, but to ensure an accurate definition, the narrow morphotype was defined as an average thallus width < 1 cm, whereas the typical morphotype was an average thallus width > 1 cm. Measurements for the two morphotypes fell within either category with no intermediates.

### 2.3 DNA extraction and microsatellite genotyping

All collected thalli were also genotyped (*n* = 115). For a detailed description of the microsatellite genotyping protocol, see Preston, Blomster, et al. (2022) (including Supplementary Materials). Microsatellite genotyping followed the aforementioned protocol exactly, although a brief summary is provided herein and in Table S1. Eight polymorphic microsatellite loci were targeted: L20, L38, L58, L85, and L94 (Engel et al., 2003) and FSP1, FSP2, and FSP3 (Perrin et al., 2007). Genomic DNA extraction was performed using the commercially available NucleoSpin® plant II DNA extraction kit (Machery‐Nagel, 740770.250) following the standard kit protocol (PL1 buffer) with 4 mg of dried apical tips, yielding concentrations between 1.4 and 118 ng/μL. DNA was diluted 1:10 in MQH_2_O with 0.5 μL being directly added to each 12.5 μL polymerase chain reaction (PCR) reaction mix. PCR reactions used OneTaq® 2X Master Mix with Standard Buffer (New England Biolabs, M0482L) or OneTaq® Hot Start 2X Master Mix with Standard Buffer (New England Biolabs, M0484L). Primers were used in multiplex PCR reactions (L38_FSP1_FSP3 and L85_L94) and as single PCR reactions. Failed multiplex reactions were rerun as single reactions. Four PCR programs were used. All programs had an initial denaturation step of 95°C 5 min and final extension step of 72°C 5 min. Loci L20 and L94_L85 had 5 cycles of touchdown 95°C 30 s, 60–55°C 30 s (−1°C\cycle), and 72°C 30 s followed by 35 cycles of 95°C 30 s, 55°C 30 s, and 30 s 72°C. Loci L38_ FSP1_FSP3 had 40 cycles of 94°C 30 s, 55°C 40 s, and 72°C 30 s. Locus L58 had 35 cycles of 94°C 30 s, 52°C 30 s, and 72°C 30 s, and locus FSP2 had 35 cycles of 94°C 30 s, 52°C 40 s, and 72°C 30 s. Samples were genotyped using panels (L20_L85_L94, L38_FSP1_FSP3, and L58_FSP2) or as singular loci on the ABI 3730 DNA analyzer in the Molecular Ecology and Systematics (MES) laboratory at the University of Helsinki.

### 2.4 Data analysis

Alleles were scored using GeneMapper 5 (Applied Biosystems™) and meticulously checked by eye. Ploidy was discerned by the maximum observed allele count for an individual sample. Consistent and repeatable reads of ≥3 alleles in the electropherograms of individual samples strongly suggested the occurrence of polyploidy (Data S3). Several validity checks, including repeat DNA extractions and PCR reactions, were performed to ensure the consistent repeatability of allele peaks for each sample in the electropherograms. Allele peaks within polyploid samples were determined to be accurate when they could be observed consistently in PCR reactions and also represented similar peak signatures to those observed in diploid samples. The majority of alleles determined in polyploid samples were also common throughout the diploid samples suggesting that spurious amplifications are unlikely. The assumption that the observation of ≥3 alleles is a result of polyploidy is supported within *Fucus* by Coyer et al. (2006). Of 115 samples, 50 were determined to have ≥3 alleles in at least one locus, inferring a potentially polyploid sample. Assigning the allele dosage of an apparent polyploid sample is problematic using microsatellite genotyping as the marker phenotype can represent multiple genotypes. For example, a triploid sample with a marker phenotype of AB may have a genotype of AAB or ABB. Consequently, we used the software Genodive version 3.05 (Meirmans, 2020) to perform data correction as in Preston, Blomster, et al. (2022). All genotyping data analyses referenced herein were performed on Genodive version 3.05 (Meirmans, 2020) unless expressly mentioned. Clonal assignment was performed using a Stepwise Mutation Model with a threshold of 0 and allocating clones specific to population. A total of 68 genotypes were assigned from 115 samples. The proportion of clones and ploidy levels were calculated manually. Pairwise population differentiation was tested using the test statistic *Rho* (Ronfort et al., 1998) with 1000 permutations. Tests were performed with all ramets per genet (115) and only a single representative ramet per clonal lineage per site (68/115). *p* values were manually corrected using Bonferroni correction. Allele frequencies were calculated for sites AS2 and AS3 and drawn in R version 4.1.2 (R Core Team, 2021). Principal component analysis (PCA) was implemented based on allele frequencies including one ramet per genet, calculated using a covariance matrix, with 1000 permutations. Estimates of individual genetic diversity for the six clonal lineages representing ≥5 ramets [C3, C12, C23, C56, C65, and C66] were calculated as follows. A diploid dataset was created from the full polyploid dataset (115) by randomly subsampling alleles from polyploid samples using Genodive (Meirmans, 2020). From this dataset, homozygosity coefficients, uncorrected homozygosity (*H*_O_) and homozygosity by locus (HL), were calculated using *Cernicalin* V.1.3.02 (Aparicio et al., 2006) for the six clonal lineages. Per chance, all six clonal lineages were diploid; consequently, the genotypes were not directly affected by the allele subsampling. All statistical tests were performed by IBM SPSS Statistics version 27 (IBM Corp, 2020). To test for differences between sites, a Kruskal–Wallis test for each morphological feature was performed. Two Mann–Whitney tests were used to determine differences in morphological features by morphotypes at site AS2 (Askö, Sweden) and by sites AS2_N and AS3 (Askö, Sweden). One‐sample *t*‐tests were used to determine if clones within the same lineage displayed similar morphological features. The test was performed for the six clonal lineages containing ≥5 ramets (C3, C12, C23, C56, C65, and C66), while the other six inadequately represented clonal lineages were excluded. PCA analysis of morphological features (thallus height, wet weight, and mean thallus width) was performed on R version 4.1.2 (R Core Team, 2021) using ggfortify (Horikoshi & Tang, 2018; Tang et al., 2016) for all samples. Samples were grouped as the six aforementioned clonal lineages, all unique MLGs, and as a single group containing all clonal multilocus genotypes with ≤5 representative samples. The relationship between ploidy and morphology was determined using a Mann–Whitney test. The groupings were diploids (samples with maximum 2 alleles at any given loci) or polyploids (samples with ≥3 alleles at any given loci). Boxplots comparing the influence of ploidy on morphological features were drawn in R version 4.1.2 (R Core Team, 2021) with significance tested using Tukey's range tests.

## 3 RESULTS

### 3.1 Genetic structure of populations

**FIGURE 2:**
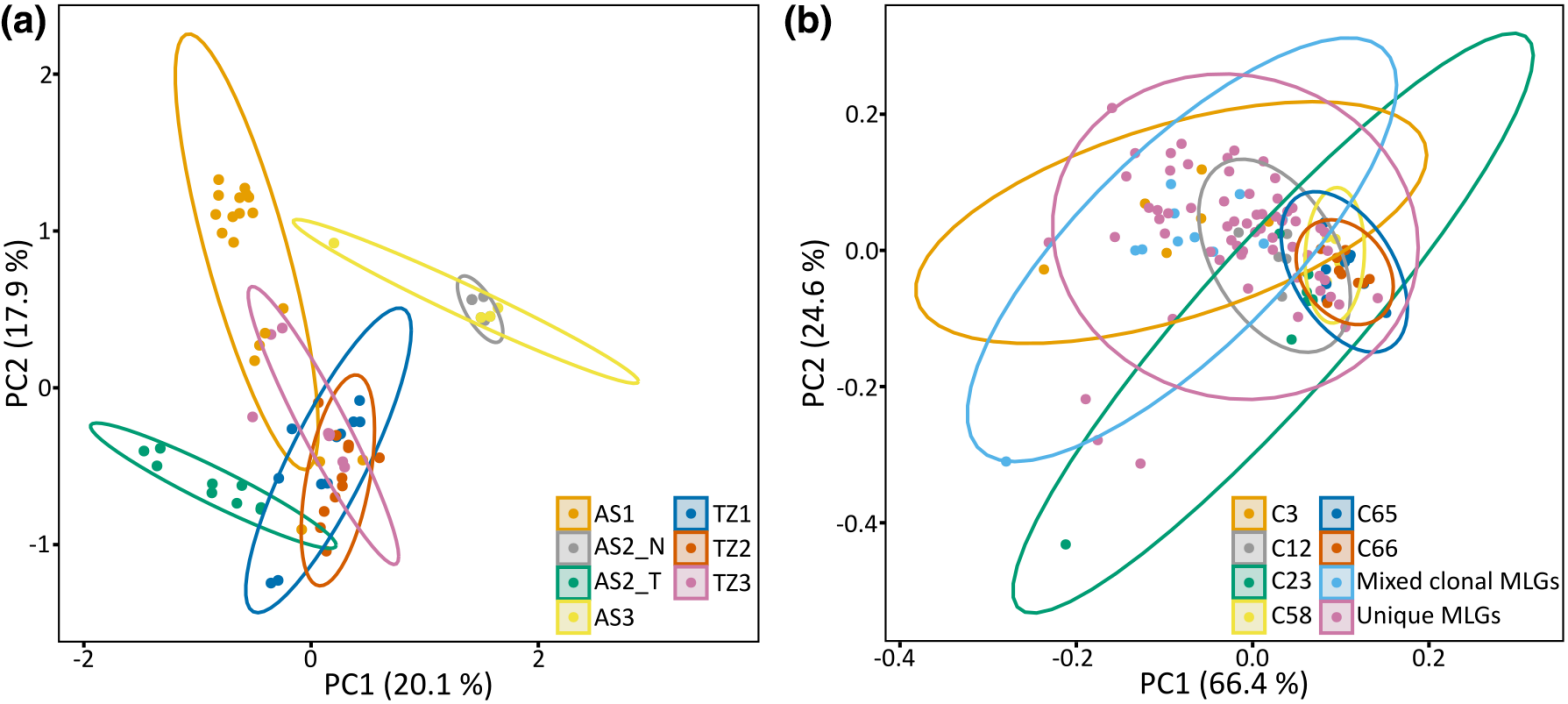
PCA representing the genetic differentiation (*Rho*_*st*_) among free‐living *Fucus vesiculosus* sites, including a single representative ramet per site (68/115) (a) and the morphological variation of all *Fucus vesiculosus* thalli (115) (b). First and second axis plotted for both PCAs. (a) Site AS2 separated by morph (N, narrow; T, typical). Abbreviations: TZ, Tvärminne; AS, Askö. (b) Analysis contains six separate clonal lineages, a single group of all unique genotypes, and a single group containing all clonal multilocus genotypes with ≤5 representative samples. Eclipses denote the distribution of thalli within the defined groups. Abbreviations: C, clonal lineage; MLGs, multilocus genotypes.

The total number of genotypes at each site ranged from 4 to 19 (Table 1). Morphotypes at AS2 represented narrow [6] and typical [9] genotypes. All sites showed multiple unique MLGs and between 1 and 4 clonal MLGs (Figure 1). No clonal MLGs were shared among sites. Unique MLGs represented a large proportion of the samples at all sites except AS3. Clonal MLGs were well represented at all sites except AS1. The two morphotypes at AS2 were represented by at least 50% unique MLGs each, with all other samples being from two morphotype‐specific clonal MLGs (Figure 1). Evidence of polyploidy was suggested at all sites (5%–80%) (Figure 1). Triploidy appeared the most commonly observed polyploidy, with only one site (TZ1) displaying tetraploidy. Comparisons between sites showed no consistent patterns in the representation of diploids or apparent polyploids. Diploidy was the dominant ploidy observed in four of the sites. Subbasin membership displayed no discernible pattern in relation to clonality or polyploidy. Diploidy appeared weakly dominant across sites within subbasin (Askö [60%, *n* = 36], Tvärminne [54%, *n* = 29]), and across all sites [57%, *n* = 65]. Genetic differentiation varied from *Rho*_*st*_ 0 to 0.7 when including a single representative ramet per genet and *Rho*_*st*_ 0 to 0.8 when including all ramets per genet (Table 2). Subbasin membership influenced genetic differentiation, with sites in Tvärminne being on average less genetically differentiated than when compared between subbasins. Genetic differentiation at Askö was highly variable with the sites showing both the highest and lowest *Rho*_*st*_ values (Table 2). Morphotypes at AS2 were highly differentiated (*Rho*_*st*_ = 0.7). The two extreme *Rho*_*st*_ values were both associated with the narrow morphotype at AS2 (AS2_N). The narrow morphotype showed high genetic differentiation when compared to all sites except for AS3 (Table 2; Figure 2a). The genetic differentiation between these two sites (AS2_N and AS3) was remarkably low (*Rho*_*st*_ = 0–0.003). The two sites shared the most common alleles in similar proportions for all loci (Data S4). Conversely, at several loci the most common alleles were not shared between the sympatric morphotypes at AS2. The first two principal components of the PCA explained 38% of the variance in the data (Figure 2a). Sites AS2_N and AS3 were separated from the other sites on the first principal component. These two sites formed a separate cluster, as supported by the elevated *Rho*_*st*_ in the pairwise distance matrix (Table 2). All other sites showed closer grouping on the first principal component with greater separation of AS1 on the second principal component. Sites from Askö showed greater separation from those within the same subbasin (except AS2_N and AS3), while all sites at Tvärminne grouped more closely. The general spread of the PCA was quite broad, representing some degree of differentiation both within and between the sites (Figure 2a) as supported by the significant *Rho*_*st*_ values (Table 2).

**TABLE 2:** Pairwise *Rho*_*st*_ genetic distance matrix for seven *Fucus vesiculosus* sites.

	AS1	AS2_N	AS2_T	AS3	TZ1	TZ2	TZ3
AS1	‐	0.643*	0.455*	0.686*	0.389*	0.508*	0.472*
AS2_N	0.583*	‐	0.754*	0.003	0.563*	0.702*	0.674*
AS2_T	0.437*	0.709*	‐	0.806*	0.398*	0.518*	0.523*
AS3	0.484*	0	0.626*	‐	0.615*	0.735*	0.716*
TZ1	0.373*	0.542*	0.375*	0.462	‐	0.299*	0.373*
TZ2	0.418*	0.587*	0.467*	0.51*	0.219*	‐	0.519*
TZ3	0.364*	0.545*	0.417*	0.412*	0.246*	0.312*	‐

Abbreviations: AS, Askö; TZ, Tvärminne. Site AS2 separated by morphotype (N, narrow; T, typical). Upper matrix analyses included all ramets per genet (115) and lower matrix analyses include a single representative ramet per genet per site (68/115). Bonferroni‐corrected *p* value significance level: <.05, *.

### 3.2 Genetic influences on morphology

Intraspecific morphological variation was evident for the species, with the sites significantly varying by thallus height (H = 42.95, *p* ≤ .001), mean thallus width (H = 61.19, *p* ≤ .001), and wet weight (H = 40.18, *p* ≤ .001). Six clonal lineages [C3, C12, C23, C56, C65, and C66] had adequate representation (≥5 ramets) to investigate morphological variation among clones (Askö [3], Tvärminne [3]). These clonal lineages had varied individual genetic diversities (H_o_ = 0.25–0.63; HL = 0.22–0.60) (Table S2). The samples within these clonal lineages showed a range of morphological features, with differences between clones within each lineage being significant in terms of all measured morphological features (Table S3). Thallus width was the most variable morphological feature between clones, with wet weight being the least. The PCA explained 90.97% of the variance in the data with two axes (Figure 2b). The first principal component determined greater dissimilarity of ramets in clonal lineages C3 (TZ1) and C23 (TZ3). All other clonal lineages showed a closer grouping (Figure 2b), indicating that ramets from these lineages are more similar in terms of morphology. The group of unique MLGs showed greater variance across first and second principal components than all clonal lineages except for the aforementioned C3 and C23 (Figure 2b).

The two distinct morphotypes at AS2 (AS2_N and AS2_T) had some of the highest levels of genetic differentiation within the whole dataset (Figure 2a, Table 2). As previously mentioned, these morphotypes were easily identifiable through visual inspection, yet we also confirm that the morphological divergence was statistically significant (Table S4a). Consequently, the morphotypes at AS2 were both genetically and morphologically divergent despite their sympatric nature. Conversely, sites AS2_N and AS3 showed little genetic differentiation and indication of morphological similarity. The two sites represent a large proportion of clones (AS2_N = 50% and AS3 = 90%), with clonal lineage C58 originating from AS2_N and clonal lineages C65 and C66 from AS3. These clonal lineages grouped closely together in the PCA indicating that morphological features were similar between these clonal lineages (Figure 2b). Supporting this, morphological features did not significantly vary between the two sites (thallus height: U = 75.0, *p* = .286; wet weight: U = 91.0, *p* = .713), except for the thallus width (mean thallus width: U = 51.5, *p* = .031) (Table S4b). Therefore, the genetic similarity between the sites is mirrored in the morphological similarity.

**FIGURE 3:**
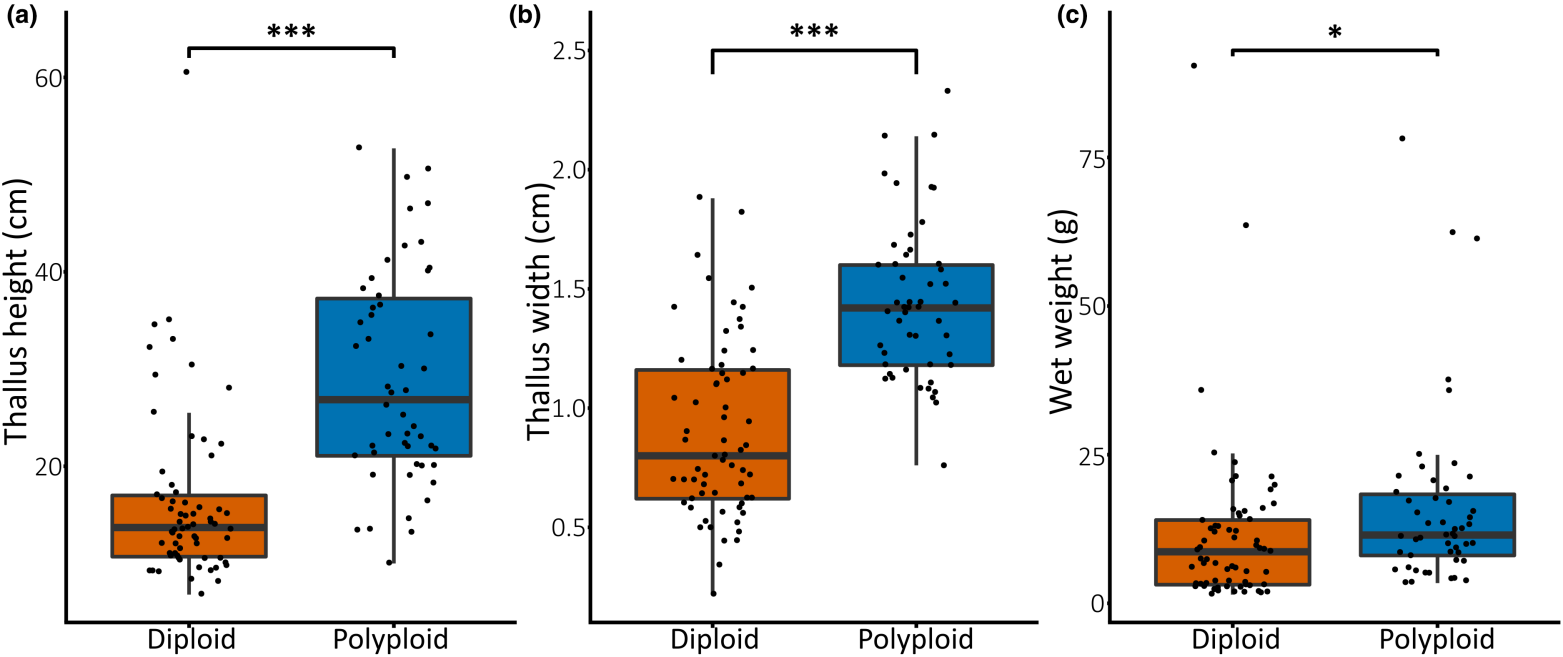
Boxplots indicating the influence of ploidy level on morphological features of *Fucus vesiculosus* thalli: thallus height (a); mean thallus width (b); wet weight (c). (*p* value significance level: *≤.05, **≤.01, ***≤.001).

The data consisted of 65 diploid and 50 polyploid samples. Correlations were identified between ploidy and morphology with apparent polyploids being significantly larger than their diploid counterparts (Table 3; Figure 3). This is shown by the Mann–Whitney mean ranks, which for all morphological variables were consistently higher in the polyploid grouping, ranging between 40 and 50 for diploids and 68 and 81 for polyploids (Table 3). The enlargement of morphology due to polyploidy is more supported in thallus height (U = 487, *p* = .000) and thallus width (U = 466, *p* = .000), while less but still significantly within wet weight (U = 1116, *p* = .004). The median thallus heights and widths were significantly dissimilar (*p* < .001) between ploidies (Figure 3a,c), while the medians for wet weight were more similar (Figure 3b). Diploid thallus height was relativity tightly grouped with several outliers. Polyploids were more loosely grouped with no outliers. The largest thallus height was recorded in a diploid sample; however, this sample was over 20 cm larger than the next largest diploid sample. This outlier does not represent the general trend in diploid thallus height. The maximum thallus height was greater for polyploids, while the minimum for each ploidy was more similar (Figure 3a). For wet weight, both ploidies were relatively tightly grouped, although both have several outliers (Figure 3b). Again, the largest recorded measure was from a diploid sample outlier. The distribution of diploid samples was skewed toward the lower scale, while polyploids are more equally distributed. Both ploidy levels displayed equal spread among thallus width, with a large range of widths being observed (Figure 3c). Maximum ranges between ploidies were similar, while minimum were more dissimilar.

**TABLE 3:** Mann–Whitney test determining differences in morphological features by ploidy level.

	Ploidy	*n*	Mean rank	Sum of ranks	Mann–Whitney U	*p* value (2‐tailed)
Height	Diploidy	65	40.49	2632.00	487.00	.000
	Polyploidy	50	80.76	4038.00		
	Total	115				
Mean thallus width	Diploidy	65	40.17	2611.00	466.00	.000
	Polyploidy	50	81.18	4059.00		
	Total	115				
Wet weight	Diploidy	65	50.16	3260.50	1115.50	.004
	Polyploidy	50	68.19	3409.50		
	Total	115				

Abbreviation: *n*, sample size.

## 4 DISCUSSION

Here, we observed several genetic influences on the morphology of free‐living *F. vesiculosus*. Overall, the relationship between genetics and morphology appears heterogeneous, yet we determine three main trends. First, thalli subjected to the same environmental conditions displayed unique morphology in correlation with genotype. This denotes that genetically distinct morphotypes have a set range of morphological traits which when subjected to the same environmental conditions do not lead to convergent morphology. We confirmed that two sympatric morphotypes had variations in their genetic stock, yet they can still be considered the same species. Second, ramets can display morphological divergence. This indicates either phenotypic plasticity, whereby the same genotype can display various morphological traits or intraclonal variation caused by genome changes during somatic growth. Third, ploidy levels appear to correspond to morphological features denoting the overall thallus size. This corresponds with the commonly observed pattern in terrestrial plants, whereby polyploids are often larger than diploid conspecifics. Consequently, we suggest that complex relationships between genetic and environmental factors generate the vast array of intraspecific morphological features observed in Baltic Sea free‐living *F. vesiculosus*. Overall, free‐living *F. vesiculosus* has the potential to exhibit high complexity through the ability to express morphological variation at both the intraspecific and intraclonal level.

### 4.1 Genotype determines morphology

In *F. vesiculosus*, genotype has been observed to influence several phenotypic features, including tolerance to warming and acidification (Al‐Janabi et al., 2016), resistance and tolerance to fouling (Honkanen & Jormalainen, 2005), and production of antiherbivory compounds (Jormalainen & Ramsay, 2009). Consequently, it is unsurprising that we determine that genotype appears to express control over morphological traits. The corresponding morphological and genetic differentiation at AS2 (i.e., AS2_N and AS2_T) determines that the morphotypes are both morphologically and genetically distinct. Therefore, we suggest that they represent two discrete ecotypes (ecologically distinct lineages) residing within a single free‐living site. However, it is perplexing that the two ecotypes remain differentiated while being within an intermingled population with no perceivable environmental barriers. Geneflow should result in the effective homogenization of the genepool, yet we observed high differentiation between the ecotypes suggesting reproductive isolation. The reproductive dynamics of free‐living populations may offer an explanation. Free‐living populations are suggested to form from thalli removed from distant populations and then be at least partially sustained through asexual reproduction within the population (Bauch, 1954; Cotton, 1912; Häyrén, 1949; Luther, 1981; Preston, Blomster, et al., 2022; Svedelius, 1901). Thus, these two ecotypes likely originate from two separate populations with distinct environmental conditions and limited connectivity, which force the development of divergent phenotypes and genotypes. The rafting pieces of thalli from each separate population may then migrate to the free‐living site, whereby they increase their representation within the site by clonal growth. As free‐living forms are surmised to rarely reproduce sexually (Bauch, 1954; Häyrén, 1949; Svedelius, 1901), barriers to gene flow are maintained through strongly restricted recombination induced by high levels of asexual reproduction. Consequently, the two ecotypes coexist within new, neutral environmental conditions, yet morphology is genetically fixed from their population of origin. This means that the two morphotypes are a result of genetic responses to two different habitats, likely geographically distant from AS2, which retain their morphological traits when supplied into the neutral free‐living habitat. Overall, the ecotypes are discrete entities with clear differences in morphology and genetics.

Further supporting that there may be an underlying genetic basis to expressed morphological traits; thalli at AS2_N and AS3 were genetically and morphologically similar. These sites were composed of varying proportions of clonal and unique MLGs with allele frequencies being remarkable similar, yet no clonal lineages were shared. Despite the geographic distance (4.3 km), these closely related genotypes formed analogous morphotypes, consequently inferring that these genotypes encode the same phenotype. Thus, it appears that genotype exerts some level of control on the morphology of *F. vesiculosus*.

It is important to note that although genotypes are associated with different phenotypes, the microsatellite markers used within this study are putatively neutral and therefore would be assumed not to be under selection. Consequently, the genotype variants should confer no fitness advantage (Stouthamer & Nunney, 2014). Thus, microsatellite genotyping can only provide partial insight into the genotype–phenotype relationship. The use of adaptive markers (i.e., genes that directly influence fitness) would be required to determine a direct link between phenotypic features and genotype (Kirk & Freeland, 2011). However, as a nonmodel organism, candidate genes and the phenotypic traits that they influence in natural populations are poorly understood in *Fucus* species.

### 4.2 Divergent morphology in genetically identical thalli

The high occurrence of clonal MLGs provides a novel situation, whereby one can examine the phenotypes of genetically identical physiological individuals in natural populations. As the ramets within clonal lineages show intraclonal variation in morphological traits, there is indication for either a potential plastic response or somatic mutations during growth. Which of these two mechanisms underlines the intraclonal variation is debatable.

Phenotypic plasticity construes that a given genotype expresses different phenotypes in different ecological settings as a result of different gene expression or gene product use in response to stimuli or inputs from the environment (Nurnberger, 2013; Pigliucci et al., 2006; West‐Eberhard, 2008). In a systematic review, 46.8% of algae were determined to show plasticity, with inducible defenses from herbivory being the primary cue for triggering plasticity, although observed plasticity in response to the environment is also common (Padilla & Savedo, 2013). Although all *F. vesiculosus* ramets within each genet were only present in single sites, environmental factors and herbivory are likely to differ across the site as well as each ramet being subjected to differing microenvironments. For example, within the Baltic Sea it is known that the rate of herbivory on *F. vesiculosus* varies with depth (Jormalainen & Ramsay, 2009). Consequently, the observed intraclonal variation may be a result of phenotypic plasticity of genetically identical ramets.

The observed intraclonal variation may also be a consequence of genome changes during somatic growth. Intraclonal variation in algae may result from several mitotic processes, including somatic mutations, intragenomic recombination, mobile genetic elements, gene duplication, and ploidy changes (Buss, 1985). As microsatellites do not represent the whole genome, some level of genomic variation within clonal lineages may be masked. Additionally, within algae mutations appear fairly common (Russell, 1986), with some resulting in alterations to the phenotype (Poore & Fagerström, 2000; van der Meer, 1981, 1990; van der Meer et al., 1984). Unlike many algae, *Fucus* spp. have a rather simplistic life history with a diplontic monophasic life cycle (de Bettignies et al., 2018) where the only haploid stages are the gametes. This limits the fixation of mutations, as most mutations are recessive meaning that the ploidy level of somatic cells thwarts selection within a clonal lineage (Klekowski, 1988). However, reproduction through clonal growth poses a far greater chance of preserving somatic mutations. In *F. vesiculosus* growth occurs through apical cells, whereby if the somatic mutation occurs in an apical cell, the mutant cell genotype will be passed on to all subsequent tissue derived from that cell resulting in genetic mosaicism (Poore & Fagerström, 2000). The resulting changes in morphology will then appear first at the tips of the thallus and as growth progresses a part of the clone develops a different phenotype (Santelices, 2001). After divergent morphological development occurs, the phenotypically varied piece may become detached from the larger thallus and form a new physiologically independent morphological variant (Santelices & Varela, 1993).

Thus, the observed morphological divergence in clonal lineages could be a result of either changes in gene expression or changes in the genome. The first would be a reversible change, while the latter is genetically fixed. It could perhaps be argued that the easiest way to determine this is to sequence either larger sections or the whole genome of each ramet. However, as a nonmodel organism, to date the complete genome of *F. vesiculosus* is unavailable. Furthermore, the understanding of genes linked to morphological traits in *Fucus* spp. is poor. Consequently, the availability of suitable genetic techniques to determine intraclonal variation within *F. vesiculosus* is currently inadequate. As frequently performed in plants, reciprocal transplant and common garden experiments to determine if changes are reversible or genetically fixed pose a more promising path (De Villemereuil et al., 2016; Linhart & Grant, 1996).

Classically, clonal organisms have been viewed as more susceptible to detrimental effects caused by changing environmental conditions. However, intraclonal variation among ramets suggests a level of adaptive potential within clonal organisms. Intraclonal variation can increase the possibility of genet survival (Santelices et al., 1995). If morphological variants correspond to improved fitness traits, this allows the genet to be able to adjust to environmental changes improving the persistence of the genet. This would be true of both phenotypic plasticity and somatic mutation. Although this concept may be particularly relevant when considering somatic mutations, as these may be inherited by subsequent generations which then offer potential for adaptive evolution in the absence of recombination (Poore & Fagerström, 2000). However, irrelevant of the true underlying mechanism causing intraclonal variation, the observation indicates that clonal free‐living *F. vesiculosus* of the Baltic Sea has the potential to adapt to changing environmental conditions. Importantly, intraclonal variation poses an ability to increase the complexity in free‐living *F. vesiculosus* despite persistent clonality.

### 4.3 Ploidy influences morphology

Polyploidy is common in many algal groups including Phaeophyceae (Goff & Coleman, 1990; Lewis, 1996; Phillips et al., 2011), with a few observations including attached *Fucus* spp. (Gómez Garreta et al., 2010) and unattached *Fucus cottonii* (determined as *F. vesiculosus*, *F. spiralis*, and/or their hybrids (Sjøtun et al., 2017), and *F. vesiculosus* (Coyer et al., 2006)). However, the documentation of potential polyploidy within the Baltic Sea is relatively recent (Preston, Blomster, et al., 2022) with numerous genetic studies focusing on *Fucus* spp. within the Baltic Sea describing diploid populations (e.g., Ardehed et al., 2016; Pereyra et al., 2009; Tatarenkov et al., 2007). The current understanding of *Fucus* polyploids, potentially deriving from either allo‐ or autopolyploidization (Coyer et al., 2006; Sjøtun et al., 2017), confounds our ability to determine the exact processes deriving polyploidy. However, stressful habitats with regard to salinity and temperature, as the Baltic Sea could be considered, have been suggested to facilitate polyploidization in *Fucus* (Sjøtun et al., 2017). Further research using appropriate techniques (e.g., microspectrofluorometry or flow cytometry) is needed to assess the accuracy of polyploidy within the Baltic Sea population; however, the genotyping data presented here strongly suggests polyploidization. The paucity of data relating to polyploidization in *Fucus* spp. limits the ability to assess how polyploidy changes ecological interactions (Segraves, 2017). However, our observed correlation between morphological traits and ploidy suggests potential consequences at a community level.

Polyploidy results in an increase in cell DNA content which has direct consequences for cell size (Müntzing, 1936; Stebbins, 1971). This occurrence, termed the Gigas effect, results in an increased cell size and consequent increase in organ and plant size (Sattler et al., 2016). The impacts of the Gigas effect on morphology are well documented in plants (Doyle & Coate, 2019; Knight et al., 2005) with the general trend resulting in polyploids having larger features than their diploid conspecifics (Porturas et al., 2019; Stebbins, 1971). However, a few exceptions in plants have also been documented (Ning et al., 2009; Segraves & Thompson, 1999; Trojak‐Goluch & Skomra, 2013; Vamosi et al., 2007), and the effect of genome size appears weaker at higher levels (e.g., tissue or organs (Knight & Beaulieu, 2008)). These exceptions twinned with the previous anecdotal observation of miniaturization in *Fucus* polyploids (Coyer et al., 2006; Sjøtun et al., 2017) indicated that *Fucus* spp. may not follow the Gigas effect. However, our observations invalidate this assumption. As free‐living *F. vesiculosus* frequently reproduces through clonal growth, the Gigas effect may have significant influences in population dynamics by enhancing the establishment success of polyploid genets through facilitating the production of more ramets and increasing ramet establishment and survival rates. Likewise, the Gigas effect can affect interactions with other organisms potentially altering the ecology of these species (Segraves & Anneberg, 2016). The interactions between the Gigas effect and community composition and functioning are likely to be complex, as although the thallus biomass is larger, the growth rate and tissue composition of polyploids will likely be altered as well (Segraves, 2017). Thus, although we appear to identify correlation between increasing ploidy and morphology, the influences on ecological interactions require further research.

However, it is important to consider that microsatellite genotyping is not an ideal method to study polyploidization. Microsatellite genotyping cannot unequivocally provide the allele dosage of an individual sample with unknown ploidy. This is because two peaks in the electropherogram (AB) may be AB if diploid, AAB or ABB if triploid, or AAAB, AABB, or ABBB if tetraploid. Similarly, a single peak (A) may represent A if haploid, AA if diploid, AAA if triploid, and AAAA if tetraploid. As polyploidy has only recently been documented in the Baltic Sea, our study worked under the a priori assumption of a diploid system. Due to this limitation, we highlight that further research using more appropriate techniques is required (e.g., microspectrofluorometry or flow cytometry). However, microsatellite genotyping does allow a loose understanding of polyploidy, as long as meticulous validity checks are implemented, because microsatellite loci are polymorphic. Consequently, detection of polyploids is possible, albeit less accurately then more specialized techniques. We emphasize that rates of polyploidy in our study are likely underestimated. Through using microsatellite genotyping, polyploids may be masked as diploids and similarly tetraploids may be masked as diploids or triploids. In our system, we assumed a tetraploid dominance of polyploids, as is the general consensus in many natural polyploid populations (Comai, 2005). Yet nearly, all polyploid samples appeared triploid. Thus, we suggest that the use of microsatellite markers has hindered the ability to assess levels of polyploidy. Although this underrepresentation of tetraploids may also translate into an overrepresentation of diploids, we suggest that diploidy is fairly common in the system due to the levels of previous research solely identifying diploidy. Thus, we feel it is appropriate to determine differences between diploids and polyploids, but any comparison between polyploid levels within our study would be erroneous.

## AUTHOR CONTRIBUTIONS

**Ivan Rodil:** Conceptualization (supporting); methodology (supporting); resources (equal); writing – review and editing (lead). **Roxana Preston:** Conceptualization (lead); data curation (lead); formal analysis (lead); funding acquisition (lead); investigation (lead); methodology (lead); project administration (lead); resources (equal); visualization (lead); writing – original draft (lead).

## CONFLICT OF INTEREST STATEMENT

The authors declare that they have no known competing financial interests or personal relationships that could have appeared to influence the work reported in this paper.

### OPEN RESEARCH BADGES

This article has earned an Open Data badge for making publicly available the digitally‐shareable data necessary to reproduce the reported results. The data is available at https://doi.org/10.6084/M9.figshare.19690930.

## Supporting information


Data S1.

## Data Availability

Microsatellite genotyping and morphology data are openly available on Figshare: https://doi.org/10.6084/M9.figshare.19690930.
