# Supplementary material for: Genetic characteristics influence the phenotype of marine macroalga Fucus vesiculosus (Phaeophyceae)
Source: Ecol Evol. 2023 Jan 31;13(2):e9788. doi: 10.1002/ece3.9788 (PMC9889845; doi:10.1002/ece3.9788)
Supplement: Supplementary file 1 — Data S1. [file ECE3-13-e9788-s001.docx]

**APPENDICES – Preston & Rodil**

**Supplementary tables**

| Supplementary table S1: Microsatellite genotyping PCR and ABI conditions. | | | | | | |
| --- | --- | --- | --- | --- | --- | --- |
| Locus | Fluorescent dye | T_a_ (^o^C) | Polymerase^(‡)^ | Primer concentration (µM) | ABI dilution | Total number of alleles observed |
| L20 | HEX | 55^(†)^ | OneTaq® Hot Start | 0.4 | 1:150; 1:200 | 14 |
| L58 | TAMRA | 52 | OneTaq® | 0.4 | 1:50 | 4 |
| L38 | FAM | 55 | OneTaq® | 0.4 | 1:120 | 8 |
| L85 | TAMRA | 55^(†)^ | OneTaq® Hot Start | 0.48 | 1:75 | 4 |
| L94 | FAM | 55^(†)^ | OneTaq® Hot Start | 0.32 | 1:75 | 3 |
| FSP1 | HEX | 55 | OneTaq® | 0.4 | 1:120 | 6 |
| FSP2 | FAM | 52 | OneTaq® | 0.4 | 1:100; 1:150 | 12 |
| FSP3 | TAMRA | 55 | OneTaq® | 0.4 | 1:120 | 12 |
| ^(†)^ touchdown PCR -1^o^C/cycle until T_a_  ^(‡)^ reactions contained 6.25 µl polymerase master mix and 2.5 µl bovine serum albumin (0.2 mg/ml) | | | | | | |

| Supplementary table S2: Individual genetic diversity for the six clonal lineages with ≥5 ramets. Abbreviations: n, sample size; H_o_, uncorrected homozygosity; HL, homozygosity by locus. | | | |
| --- | --- | --- | --- |
| Clonal lineage | n | H_o_ | HL |
| C3 | 6 | 0.50 | 0.52 |
| C12 | 9 | 0.38 | 0.36 |
| C23 | 9 | 0.25 | 0.22 |
| C58 | 5 | 0.50 | 0.51 |
| C65 | 9 | 0.63 | 0.60 |
| C66 | 9 | 0.50 | 0.46 |

| Supplementary table S3: One-sample t-tests determining differences in morphological variables for each clonal multilocus genotype (≥5 samples). Abbreviations: df, degrees of freedom; n, sample size. | | | | | | | |
| --- | --- | --- | --- | --- | --- | --- | --- |
| Genotype | Site of origin | n | Mean | Standard deviation | df | T | P value (2-tailed) |
| Thallus height | | | | | | | |
| C3 | TZ1 | 6 | 33.67 | 14.34 | 5 | 5.75 | 0.002 |
| C12 | TZ2 | 9 | 12.36 | 1.53 | 8 | 24.18 | 0.000 |
| C23 | TZ3 | 9 | 18.43 | 6.85 | 8 | 8.07 | 0.000 |
| C58 | AS2 | 5 | 11.80 | 2.33 | 4 | 11.32 | 0.000 |
| C65 | AS3 | 9 | 11.30 | 2.82 | 8 | 12.01 | 0.000 |
| C66 | AS3 | 9 | 11.48 | 2.65 | 8 | 12.98 | 0.000 |
| Wet weight | | | | | | | |
| C3 | TZ1 | 6 | 19.040 | 10.46 | 5 | 4.46 | 0.007 |
| C12 | TZ2 | 9 | 16.11 | 5.30 | 8 | 9.12 | <0.001 |
| C23 | TZ3 | 9 | 27.29 | 29.08 | 8 | 2.82 | 0.023 |
| C58 | AS2 | 5 | 3.21 | 2.02 | 4 | 3.56 | 0.024 |
| C65 | AS3 | 9 | 3.94 | 2.68 | 8 | 4.42 | 0.002 |
| C66 | AS3 | 9 | 3.79 | 2.55 | 8 | 4.46 | 0.002 |
| Mean thallus width | | | | | | | |
| C3 | TZ1 | 6 | 1.56 | 0.28 | 5 | 13.44 | <0.001 |
| C12 | TZ2 | 9 | 1.09 | 0.24 | 8 | 13.39 | <0.001 |
| C23 | TZ3 | 9 | 0.83 | 0.23 | 8 | 10.61 | <0.001 |
| C58 | AS2 | 5 | 0.75 | 0.11 | 4 | 15.67 | <0.001 |
| C65 | AS3 | 9 | 0.62 | 0.22 | 8 | 8.50 | <0.001 |
| C66 | AS3 | 9 | 0.62 | 0.12 | 8 | 15.04 | <0.001 |

| Supplementary table S4: Mann–Whitney test determining differences in morphological variables by morphotype at site AS2 [Askö, Sweden] (A) and by sites AS2_N and AS3 [Askö, Sweden] (B). Abbreviations: n, sample size. | | | | | | |
| --- | --- | --- | --- | --- | --- | --- |
| A |  |  |  |  |  |  |
|  | Morphotype | n | Mean Rank | Sum of Ranks | Mann–Whitney U | P value (2-tailed) |
| Thallus height | Typical | 10 | 14.90 | 149.00 | 6.00 | 0.000 |
|  | Narrow | 10 | 6.10 | 61.00 |  |  |
|  | Total | 20 |  |  |  |  |
| Wet weight | Typical | 10 | 14.60 | 146.00 | 9.00 | 0.001 |
|  | Narrow | 10 | 6.40 | 64.00 |  |  |
|  | Total | 20 |  |  |  |  |
| Mean thallus width | Typical | 10 | 15.50 | 155.00 | 0.00 | 0.000 |
|  | Narrow | 10 | 5.50 | 55.00 |  |  |
|  | Total | 20 |  |  |  |  |
|  |  |  |  |  |  |  |
| B |  |  |  |  |  |  |
|  | Site | n | Mean Rank | Sum of Ranks | Mann–Whitney U | P value (2-tailed) |
| Thallus height | AS2_N | 10 | 18 | 180.00 | 75.00 | 0.286 |
|  | AS3 | 20 | 14.25 | 285.00 |  |  |
|  | Total | 30 |  |  |  |  |
| Wet weight | AS2_N | 10 | 16.40 | 164.00 | 91.00 | 0.713 |
|  | AS3 | 20 | 15.05 | 301.00 |  |  |
|  | Total | 30 |  |  |  |  |
| Mean thallus width | AS2_N | 10 | 20.35 | 203.50 | 51.500 | 0.031 |
|  | AS3 | 20 | 13.08 | 261.50 |  |  |
|  | Total | 30 |  |  |  |  |

**Supplementary Data**

| Supplementary data S1: Examples of the surrounding environment and *Phragmites australis* reed beds at sites (A) AS1 (B) AS2. | |
| --- | --- |
| A  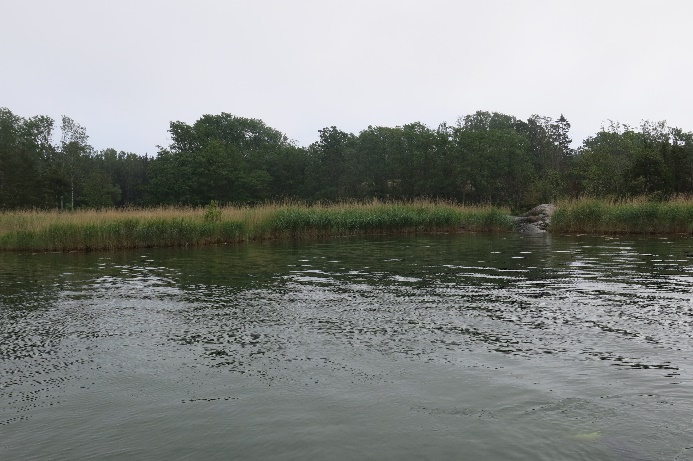 | B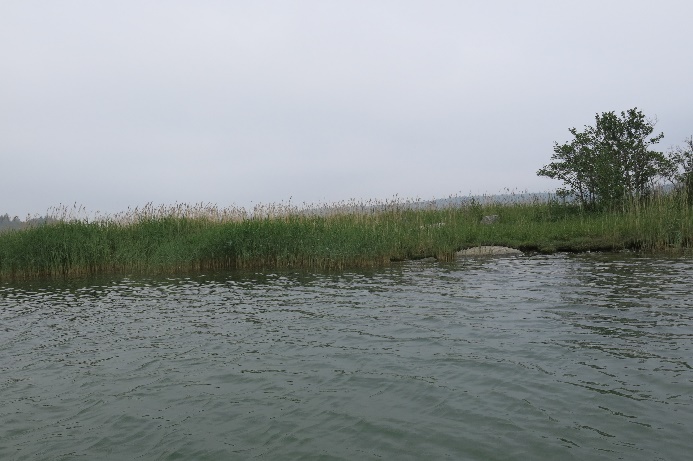 |

| Supplementary data S2: Examples of the two morphotypes observed at AS2 [Askö]. Herbarium specimens of the typical (A) and narrow (B) morphotypes; fresh specimens of the typical (C) and narrow (D) morphotypes. Scale bars represent 2.5 cm. Site AS2 was within the typical range for the recorded environmental measures (salinity/depth) observed for all sites. No discernible environmental conditions were specific to this site. The reason for sympatry at this site is unknown. | |
| --- | --- |
| A  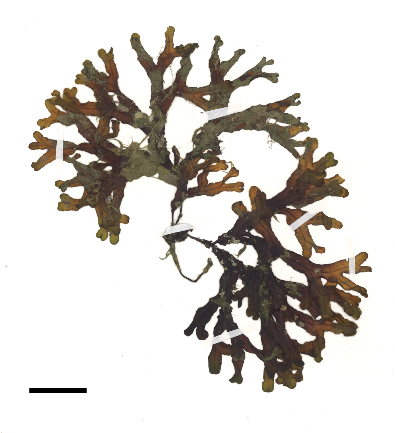 | B  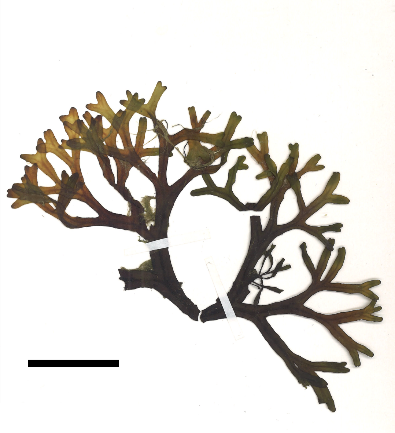 |
| 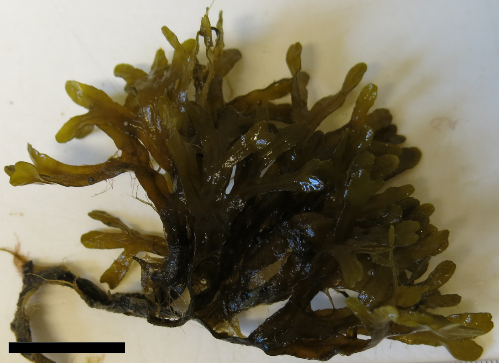C | 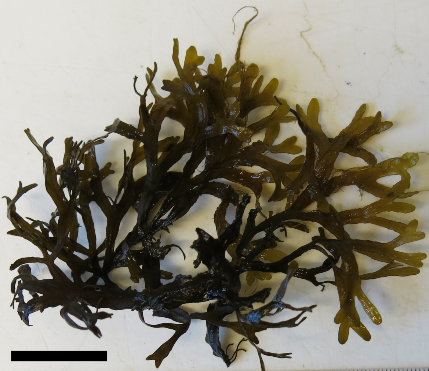D |
| Images A and B provided by Ellen Schagerström and Susanne Qvarfordt. Images C and D are author’s own. | |

| Supplementary data S3: Example microsatellite electropherogram traces for loci L38 (A) and FSP2 (B). The observed genotypes represented in these example traces were found in 18 (L38) and 7 (FSP2) samples. Five additional distinct polyploid genotypes were represented by each loci (electropherograms traces not shown). | |
| --- | --- |
| **A**  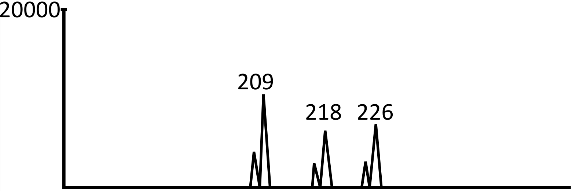 | **B**  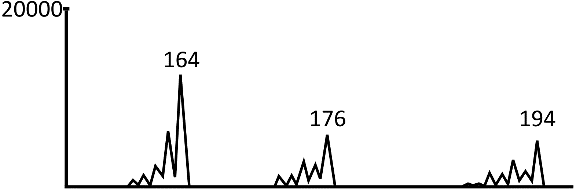 |

| Supplementary data S4: Allele frequencies for sites AS2 and AS3 including all ramets [40] (A) and a single ramet per genet per population [19/40] (B). AS2 separated into typical and narrow morphotypes. Colour key: AS2_T, orange; AS2_N, blue; AS3, pink. |
| --- |
| **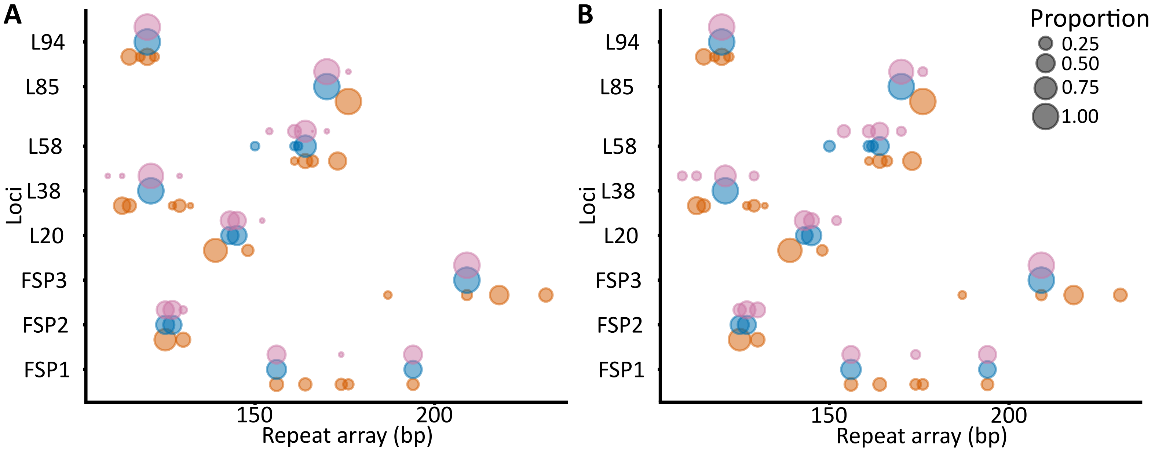** |
